# Supplementary material for: Mir324 knockout regulates the structure of dendritic spines and impairs hippocampal long-term potentiation
Source: Sci Rep. 2023 Dec 8;13:21919. doi: 10.1038/s41598-023-49134-w (PMC10713680; doi:10.1038/s41598-023-49134-w)
Supplement: Supplementary file 1 — Supplementary Figures. [file 41598_2023_49134_MOESM1_ESM.pdf]

Supplementary Data For

MiR-324-5p regulates the structure of dendritic spines and impairs hippocampal long-term potentiation

Parkins, Emma V[1,2]; Brager, Darrin H[3]; Rymer, Jeffrey K[2]; Burwinkel, John M[2]; Rojas, Diego[2]; Tiwari, Durgesh[2,4]; Hu, Yueh-Chiang[4,5,6]; Gross, Christina[1,2,4*]

[1]University of Cincinnati Neuroscience Graduate Program, Cincinnati, Ohio 45229, USA

[2]Division of Neurology, Cincinnati Children's Hospital Medical Center, Cincinnati, Ohio 45229, USA

[3]Center for Learning and Memory, Department of Neuroscience, The University of Texas at Austin, Texas 78712, USA

[4]Department of Pediatrics, University of Cincinnati College of Medicine, Cincinnati, Ohio 45229, USA

[5]Transgenic Animal and Genome Editing Core Facility, Cincinnati Children's Hospital Medical Center, Cincinnati, Ohio 45229, USA

[6]Division of Developmental Biology, Cincinnati Children's Hospital Medical Center, Cincinnati, Ohio 45229, USA

***Correspondence should be addressed to:**

Christina Gross: christina.gross@cchmc.org

Location R, R2.407, 3333 Burnet Avenue, Cincinnati, Ohio 45229

Content:

Supplementary Figure S1

Supplementary Figure S2

Supplementary Figure S1

CTGACTATGCCTCCTCGCATCCCCTAGGGCATTGGTGTAAAG
CTGGAGA*CCCACTGCCCCAGGTGCTGCTGG*GGGTTGTAGTC

sgRNA-targeted sequence (in red): CTAGGGCATTGGTGTAAAGC

Deleted sequence (*Mir324* KO; highlighted): CCCCTAGGGCAT

miR-324-5p (underlined): CGCATCCCCTAGGGCATTGGTG

miR-324-3p (underlined and italicized): *CCCACTGCCCCAGGTGCTGCTGG*

Supplementary Figure S1: Mir324 sequence. The *Mir324* gene is 83 base pairs long. In the above sequence, the underlined portion indicates the 22-nucleotide sequence of mature miR-324-5p. The underlined and italicized portion shows the sequence of miR-324-3p. The deleted portion for *Mir324* knockout (KO) is highlighted in gray. In red, the region targeted by the sgRNA for generating *Mir324* KO mice is shown.

Supplementary Figure S2

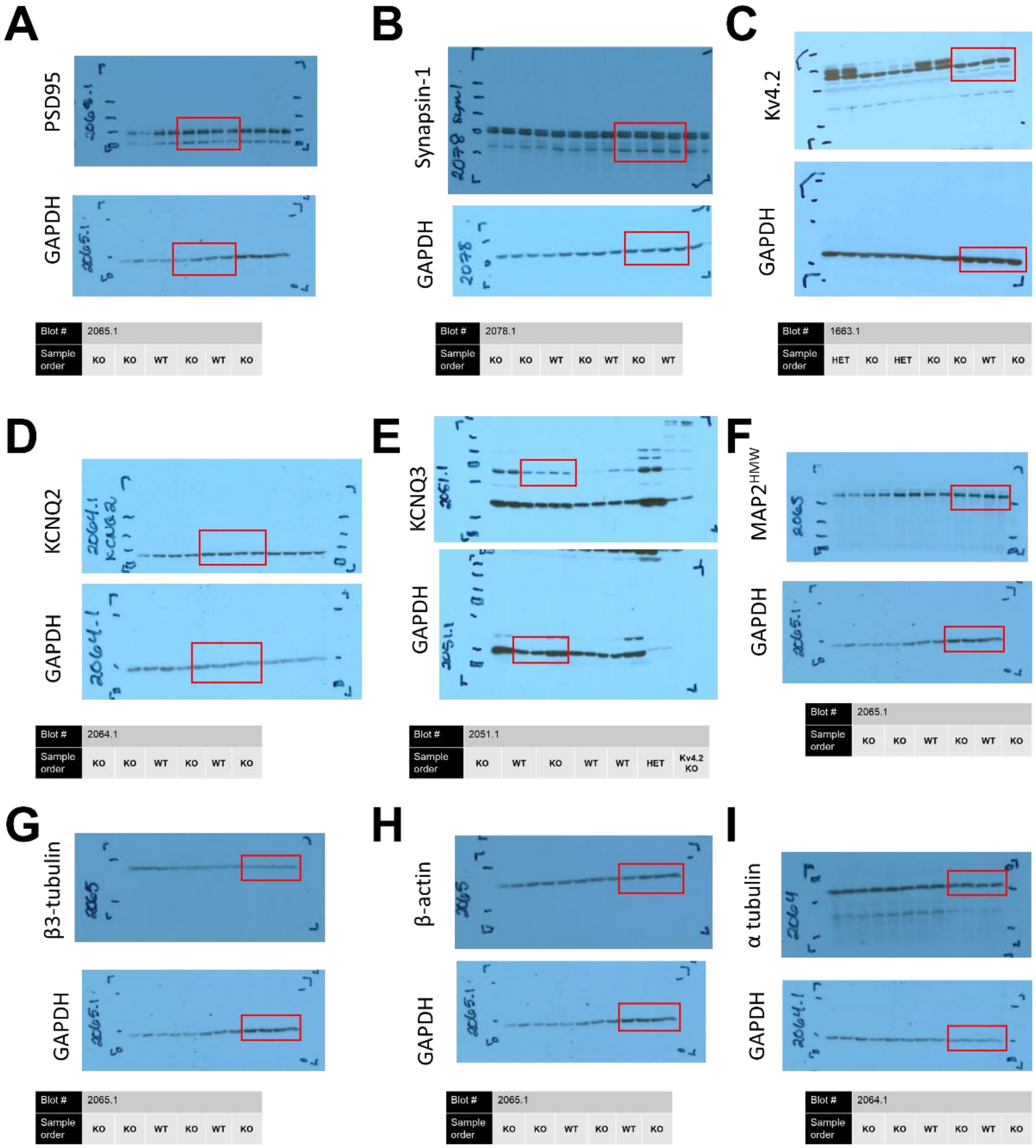

Supplementary Figure S2: Western blot images. Uncropped images of complete western blots shown in Figures 2 and 6. Lanes used for representative images are enclosed in red boxes. The proteins are (**A**) PSD95, (**B**) synapsin-1, (**C**) Kv4.2, (**D**) KCNQ2, (**E**) KCNQ3, (**F**) MAP2^{HMW}, (**G**) β3-tubulin, (**H**) β-actin, and (**I**) α-tubulin. Note that several proteins were measured on the same blot. Each hippocampal tissue sample is loaded in duplicate. Blot number and order of genotypes are listed in tables below the blot images.
